# Monitoring obesity prevalence in the United States through bookmarking activities in online food portals

**DOI:** 10.1371/journal.pone.0179144

**Published:** 2017-06-21

**Authors:** Christoph Trattner, Denis Parra, David Elsweiler

**Affiliations:** 1 Department of New Media Technology, MODUL University Vienna, Vienna, Austria; 2 Departamento de Ciencia de la Computación, Pontificia Universidad Católica de Chile, Santiago, Chile; 3 Chair for Information Science - I:IMSK, University of Regensburg, Regensburg, Germany; Cinvestav-Merida, MEXICO

## Abstract

Studying the impact of food consumption on people’s health is a serious matter for its implications on public policy, but it has traditionally been a slow process since it requires information gathered through expensive collection processes such as surveys, census and systematic reviews of research articles. We argue that this process could be supported and hastened using data collected via online social networks. In this work we investigate the relationships between the online traces left behind by users of a large US online food community and the prevalence of obesity in 47 states and 311 counties in the US. Using data associated with the recipes bookmarked over an 9-year period by 144,839 users of the Allrecipes.com food website residing throughout the US, several hierarchical regression models are created to (i) shed light on these relations and (ii) establish their magnitude. The results of our analysis provide strong evidence that bookmarking activities on recipes in online food communities can provide a signal allowing food and health related issues, such as obesity to be better understood and monitored. We discover that higher fat and sugar content in bookmarked recipes is associated with higher rates of obesity. The dataset is complicated, but strong temporal and geographical trends are identifiable. We show the importance of accounting for these trends in the modeling process.

## Introduction

Learning about the nutritional habits and corresponding health related issues of a population plays an important part in determining a nation’s public health policy, but it is also a slow and expensive process. Typical methods employed include telephone interviews and surveys [1–3]. Difficulties with such approaches include that they are not only cost intensive (as they involve a lot of people in the process) but also that considerable time is required to collect and process the data. For instance, the Centers for Disease Control and Prevention (CDC) [4] of the US government conduct several thousand telephone surveys every year to, amongst other things, learn about obesity and diabetes cases. It is common for the processing time of these surveys to take several months and even more time is required before the data is available to the general public. The obesity and diabetes prevalence data collected by the CDC on state and county level is, for example, at the time of writing (June 2016), only available up until the year 2012. Another problem with this kind of approach is that the results are open to several methodological biases, including subjective responses (i.e. participants present their interpretation of reality and not necessarily the objective truth) [5], memory biases (i.e. participants have difficulty accurately remembering autobiographical behavior) [6] and participants tend to present themselves in socially desirable ways [7]. The results are moreover heavily reliant on good sampling and even then must be carefully analysed because some methods can be more accurate for some subjects than others [8].

To address such issues computational approaches using naturally created user data are often mooted as a complementary data collection method [5, 9] because they allow almost real-time access to behavioural trends. This approach has been applied to, for example, track flu trends using digital traces in the form of search terms left behind by people using Web search engines [10], model stockmarket trends based on social-media content [11] and predict election outcomes using social-media posts as a barometer of public opinion [12].

**Objective**. While much work has been done to explore the utility of digital behavior traces in the context of diverse prediction problems (e.g. flu epidemics, stock market trends and election outcomes), less research has been performed with respect to understanding how similar online traces can be used to model and predict nutritional patterns and related health issues, such as obesity rates. To contribute to knowledge in this area, in this paper we analyse patterns in the traces left behind by users while they browse the Web and compare these statistically with health trend statistics in the United States. More specifically, we investigate whether or not relationships exist between online traces in the form of bookmarked recipes from the online food community platform Allrecipes.com and health problems, specifically the incidence of obesity in various geographical regions of the United States.

**Impact**. The presented research is important for several reasons. First, food is not only a driver for how good we feel on a daily basis [13], but also how healthy we are [14]. According to the World Health Organization (WHO) around 80% of cases of heart disease, strokes and type 2 diabetes could be avoided if people would implement a healthier diet [15]. Furthermore, according to the WHO, within the last three decades the numbers of people classified as being overweight or clinically obese have increased dramatically (especially in younger generations) resulting in a cost of approx. 81 billion EUR per year to help people with chronic diseases related to unhealthy eating habits [16]. Identifying predictors of health problems is one issue. Another is to obtain timely access to trends—something which current survey-based methods often fail to deliver and for which we see approaches, such as those we present here, as a complementary tool to support researchers and governmental entities in their work.

**Research Questions**. To drive our research we have defined the following three high-level research questions:

- *RQ1*. To what extent do the nutritional properties of bookmarked recipes on Allrecipes.com correlate with obesity levels in the US?
- *RQ2*. To what extent can temporal or geographical factors help in explaining obesity patterns?
- *RQ3*. To what extent can these factors be modeled to explain the variance in obesity rates across the US?

**Outline**. In the following sections we will review appropriate background literature, introduce the datasets and methodology chosen to address our research questions, and present and discuss the results of our study. Finally, we draw conclusions, discuss the limitations of our study and propose future research directions.

## Background

Our study contributes to a growing body of work using digital traces derived from using the World Wide Web as a means to monitor, understand or predict health-related activity. In the following two sub-sections we overview relevant related research. First, we summarise past efforts to use digital behavior as a means of understanding other human behaviors, problems and epidemics. In a second step, we focus on nutritional aspects related to our analyses by summarizing work from the nutritional science literature. We use this knowledge to guide our analyses and interpret the findings.

### Digital traces as an epidemiological tool

An early example showing how users’ online digital traces can be used to monitor and predict public health is the work of Polgreen and colleagues [17] who investigated the potential of influenza related queries submitted to the Yahoo! search engine to predict influenza cases and deaths in the US. Later work following a similar approach, but using Google search queries, formed the basis for the well-known Google flu tracking service [10]. More recent research has used the linguistic properties of social media content to track smoking [18], the use of alcohol [19] and other drugs [20], as well as life satisfaction [21, 22] and mental illnesses, such as depression [23]. Two comprehensive overview publications for these kinds of studies are [24] and [25].

While viruses such as H1N1 (flu) and mental illness cases have received considerable attention, relatively little research has focused on using digital traces to understand nutritional habits or health related issues such as obesity. From what we know about how people make food choices e.g. [26] we know this is a complex, multi-faceted process, influenced by biological, personal and socio-economical factors. Yet for the majority of people, aspects of taste and sensory appeal seem to be the drivers for decisions, followed by health concerns, nutritional value, and price [27], meaning that the decisions can be modeled using relatively simple heuristics [28]. All of this suggests that data regarding food preferences established via interactions with online recipes may shed light on food related, socio-economic problems, such as obesity.

Work in this direction includes West et al.’s [29] analysis of a large corpus of Microsoft Bing search-logs to determine the extent to which people search and access information in the online food community website Allrecipes.com. They discovered that people’s searches for food on the Web follow weekly and yearly trends, that they significantly differ between states and that there is a correlation between searches for food online and heart attacks recorded in the US. Similar trends were also observed by Wagner and colleagues [30] and Trattner et al. [14] using data collected via the German online food portal Kochbar.de. Said & Bellogin [31] analyzed a crawl of Allrecipes.com and using simple statistical techniques showed that there are statistically significant differences between states in the US on an ingredient level. Twitter has also been used as a basis to understand food behavior. Fried and others [32] and Abbar and others [33] both found correlations between food mentions on Twitter and health related issues such as diabetes and obesity in the US. Moreover correlations have been found between FourSquare check-in patterns at fast food restaurants and obesity patterns in the US [34]. De Choudhury et al. [35] showed that digital traces left behind by users on the social platform Instagram and poor online food consumption patterns are correlated with so-called “food deserts” (unhealthy regions) in the US. All of these analyses focused on the relationship between the occurrence of certain food types or ingredients perceived as being unhealthy and health-related metrics. Recent research has investigated how digital traces, predominately search queries, can be used to detect life-threatening diseases, such as cancer [36].

### Relevant nutritional science research

Nutritional science and epidemiology have contributed considerable knowledge with respect to how food consumption patterns relate to public health. Based on this knowledge, a technical report by the WHO expert group on diet, nutrition and prevention of chronic diseases [37] reports on the extent to which macro nutrients impact on public health, recommending that intake of sugar- and energy-dense foods should be limited to reduce the likelihood of suffering from obesity. For type-2 diabetes the recommendation is to reduce the saturated and total fats consumed. Similar findings and recommendations are reported by Gross and colleagues [38] based a longitudinal ecologic correlation study on nutrient consumption in the United States between 1909 and 1997 obtained from the US Department of Agriculture. In their work, with the help of a multivariate nutrient-density model, they discovered that corn syrup (sugar) was positively associated with the prevalence of type 2 diabetes. Fiber however, was negatively associated with the prevalence of type 2 diabetes. For protein and fat, no associations with type 2 diabetes were found, a result similar to that obtained by Meyer et al. [39] in 2000.

Van Dam and Seidell [40] provide an overview of research studying the link between obesity and the intake of certain micro nutrients. Somewhat mirroring the recommendations of the WHO, they found that sugar consumption is associated with weight gain and that fiber is associated with a lesser degree of weight gain. For further studies in this context, we refer the reader to the already cited WHO technical report [37], as well as to the database of the American journal of clinical nutrition [41].

### Differences to previous research

Our work extends previous computational research approaches in three distinct ways: (i) First, in contrast to previous work, we do not look at specific food types or specific ingredients liked by users, but rather rely on macro-nutritional properties of the kind of food people prefer (i.e. bookmark). This provides the potential for a different, richer picture of the factors affecting people’s health, perhaps building bridges to research from nutritional science and epidemiology. (ii) Second, we do not limit our study to one single snapshot of the data. All previous studies rely on data that reflects online food interactions within a single, short time frame of 1–3 months. We, instead, provide insights over a 9-year period. This allows us not only to show that the relationships are stable over time, but also to improve the modeling process by incorporating temporal aspects. This is important because there are several temporal trends in both bookmarking behavior and the incidence of obesity—we show these trends globally and across states and counties. (iii) Finally, there are methodological differences between our study and previous investigations in this area: We go beyond previous work, which offered simple correlation analyses between digital traces (e.g. the caloric values of foods mentioned in tweets per each state and obesity rate in the state), by using multi-level regression modeling approaches to understand the complex relationships between the micro nutritional properties of recipes and obesity levels at a user, county and state level over time.

## Materials

Our study relies on two datasets: (i) the US census data for diabetes and obesity prevalence (ii) and a crawl of the US online food community platform Allrecipes.com [42]. Allrecipes.com is just one of many online recipe portals. Others popular sites include food.com, epicurious.com, yummly.com and cooks.com. We chose Allrecipes.com firstly because, at the time of writing, it claims to be the world’s largest food-focused social network. The site has a community of 40 million users accessing 3 billion recipes annually across 24 countries [43]. This claim has been corroborated by services such as Alexa.com and eBizMBA, both of which rank Allrecipes.com as the most popular recipe website [44]. This means we had access to a large data set for our analyses. A second and perhaps more important justification is that, in contrast to the other sites listed above, which tend to promote aspirational dishes, such as those created by professional chefs, the recipes on Allrecipes.com reflect what ordinary people [in the United States] actually eat [45]. This is echoed by the ingredients in dishes and the language used to describe them. One commentator who analysed the nature of Allrecipes.com recipes stated that “What’s remarkable isn’t so much Allrecipes’ dominance but how distant the site feels from the food conversation in the media” [45]. The authentic everyday nature of the recipes on the site, makes Allrecipes.com the perfect data source for our analyses.

The US census data is provided by the CDC [4] and gives an yearly overview of obesity incidence in the US at state and county level. The data we used in our analysis reports the percentage of obesity incidence in each state and county and is available for the years 2004 until 2012.

In order to obtain digital traces left behind by users while using the online community platform Allrecipes.com, a standard Web crawler was implemented to mine recipes uploaded by the users of the platform and discover, which recipes were bookmarked by which users. The crawling of the platform was performed between 20^th^ and 24^th^ of July, 2015 and located 242,113 recipes published by 62,100 users between the years 2000 and 2015. Additionally, we obtained 17,817,462 recipe bookmarks from 144,839 users for the same time period.

Although we do not have any formal agreement with Allrecipes.com to collect and use the data, we were careful to collect and process the data in an ethical manner, such that the Terms of Service Agreement were respected. To do so we took the following steps: 1) we made sure to respect robots.txt and impose minimal load on Allrecipes.com during our crawls. 2) we did not affect the users of the site since we did not create or manipulate (e.g. rate or annotate) any recipes or interact with the users in any way. 3) we endeavoured to adhere to the Allrecipes.com Terms of Service Agreement, which permits the crawling of the data to create publicly available searchable indices of the materials. We are in the process of making such an index available publicly, which will allow the results of the analyses presented here to explored interactively. Not only will users of the index be able to search for and access popular and highly rated recipes by geographical region, but they will be able compare census health statistics and recipes both visually and numerically in order to better understand the relationships between the two.

## Methodology

To achieve our goals of identifying and quantifying the relationships between the macro-nutritional properties of bookmarked recipes and obesity levels, we perform two analyses: In a first step, we conduct a correlation analysis to identify trends as well as to detect issues of multicollinearity which can lead to problems in deriving and interpreting regression models. We then build on these results using a multi-level regression analysis, which is necessary in order to model relationships considering the hierarchical and longitudinal characteristics of the data.

### Data pre-processing

Performing these analyses required a number of important pre-processing steps. First, we restricted the bookmarks to the same time periods as provided by the US census data. Next, we restricted the dataset further to include only the recipes for which full nutritional information was available. Although recipes in Allrecipes.com are peer-reviewed and macro nutritional facts are generated using the ESHA [46] research database, not all nutrition properties can be calculated as some of the ingredients do not match with the ESHA database. For our analysis, we relied only on those recipes for which all ingredients were present in the ESHA food database (out of 242,113 originally crawled recipes 58,263 had nutrition information available). We chose to focus on “fat”, “saturated fat”, “sugar” and “sodium” (measured in 100g per recipe) because they allow us to measure the healthiness of a recipe according to international standards introduced in 2007 by The Food Standard Agancy (FSA) [47].

Following the procedure described in Howard et al.’s paper [48], for each meal we calculated the nutritional content per portion by dividing the total content by the number of portions in the meal. This was done to obtain a so-called FSA health score that measures on a discrete scale the extent to which a recipe is healthy or probably unhealthy [47]. The FSA front of package labeling system introduced by the Food Standard Agancy (FSA) [47] relates to 4 macro-nutrients (sugar, sodium, fat and saturated fat). The scale is green (healthy), amber and red (unhealthy). In order to derive a single metric we follow the procedure of Sacks et al. [49] who first assign an integer value to each color (green = 1, amber = 2 and red = 3) then sum the scores for each macro-nutrient resulting in a final range from 4 (very healthy recipe) to 12 (very unhealthy recipe). We use this metric throughout our analysis to determine the healthiness of recipes and we refer to it as “FSA score”.

It was necessary to map users of Allrecipes.com to states and counties in the US. This was possible because users in Allrecipes.com typically provide information about their current home location. For the purpose of our study, we considered only users who could be objectively mapped to a state and a county in the US. This was done with the help of the Google Places Web API [50].

We continued to transform the data by calculating, for each US state and county, the mean value for each nutritional property (fat, saturated fat, sugar and sodium) for recipes bookmarked by users residing in that state. From these values we derived the FSA score representing the healthiness the recipes on average. This allowed us to create yearly datasets that contain obesity incidence level at state and county level as well as the corresponding interaction, macro-nutritional properties and FSA scores.

Finally, to remove noise from the dataset we only considered those observations where for a specific year the county had at least 30 Allrecipes.com users. Although this produced a dataset with imbalanced observations (some counties are only present in later years where the number of users increased beyond 30), the regression modeling approach used to analyze the data is suited to this condition.

30 users was chosen as a cut-off point to balance the need of having a representative number of users for each county without overly restricting the data to highly populated geographical locations (e.g. cities) and counties with few problems with obesity. The dataset contains a general bias whereby fewer users reside in locations with the worst obesity situations. Again our statistical analyses are robust to this problem. The overall descriptive statistics of our dataset for the years 2004 to 2012 can be viewed in Table 1. The dataset can be obtained via the supplementary information, see S1 File.

**Table 1:** Basic statistics of the Allrecipes.com dataset with at least 30 users per county.

Year	Num. Users	Num. Bookmarks	Num. Recipes	Num. Counties	Num. States
2004	1348	29,827	1491	25	13
2005	3185	63,512	2210	54	25
2006	7149	185,251	4964	99	36
2007	10,803	270,835	6850	135	40
2008	17,873	500,063	10,227	193	43
2009	21,644	625,661	12,077	225	47
2010	27,331	910,918	15,442	256	46
2011	29,004	933,521	15,351	266	47
2012	26,093	656,364	12,738	244	47

### The statistical analysis

It is common to precede a regression modeling procedure with an exploratory data analysis to understand the data distribution, identify potential outliers and detect issues which violate the assumptions such models make. A good example is the presence of strong multicolinearity between predictors. We report EDA results by presenting a correlation analysis (Pearson correlation) considering the variables obtained from the traces extracted via Allrecipes.com (bookmarks, nutritional information from bookmarked recipes, the FSA health score) and also from the census data (obesity prevalence levels).

Moving beyond the approaches applied in previous research efforts (e.g. [29, 33, 51], we perform a multi-level regression analysis to investigate the relationships between the predictors in more detail. A standard multiple regression modeling approach would be inappropriate because the hierarchical structure of our dataset violates the assumption of independence of the observations. Our data on obesity incidence, as well as the nutritional data of bookmarked recipes stems from different counties all over the US. The dataset, moreover, has repeated observations over time (from 2004 to 2012) and we know the nested geographical structure of counties within states. Using this geographical, temporal and nutritional information we built several multilevel regression models in order to discover how these factors relate to obesity.

**Fig 1:**
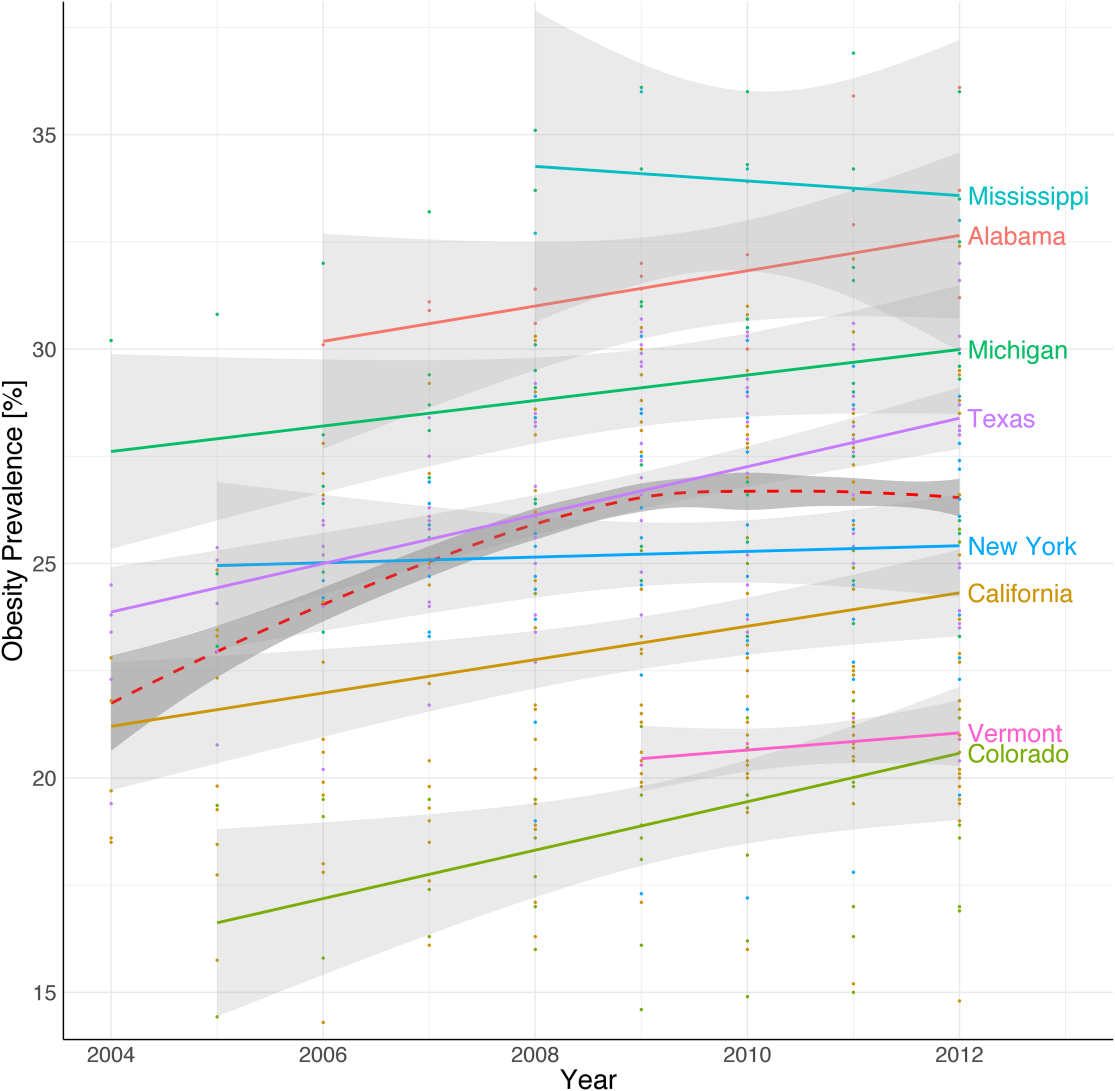
Linear trends of fat as a function of time (2004–2012) for selected states in the US. The plots show a variety of intercepts and trends (slopes) over time. The general aggregated trend is shown with a dashed line.

**Fig 2:**
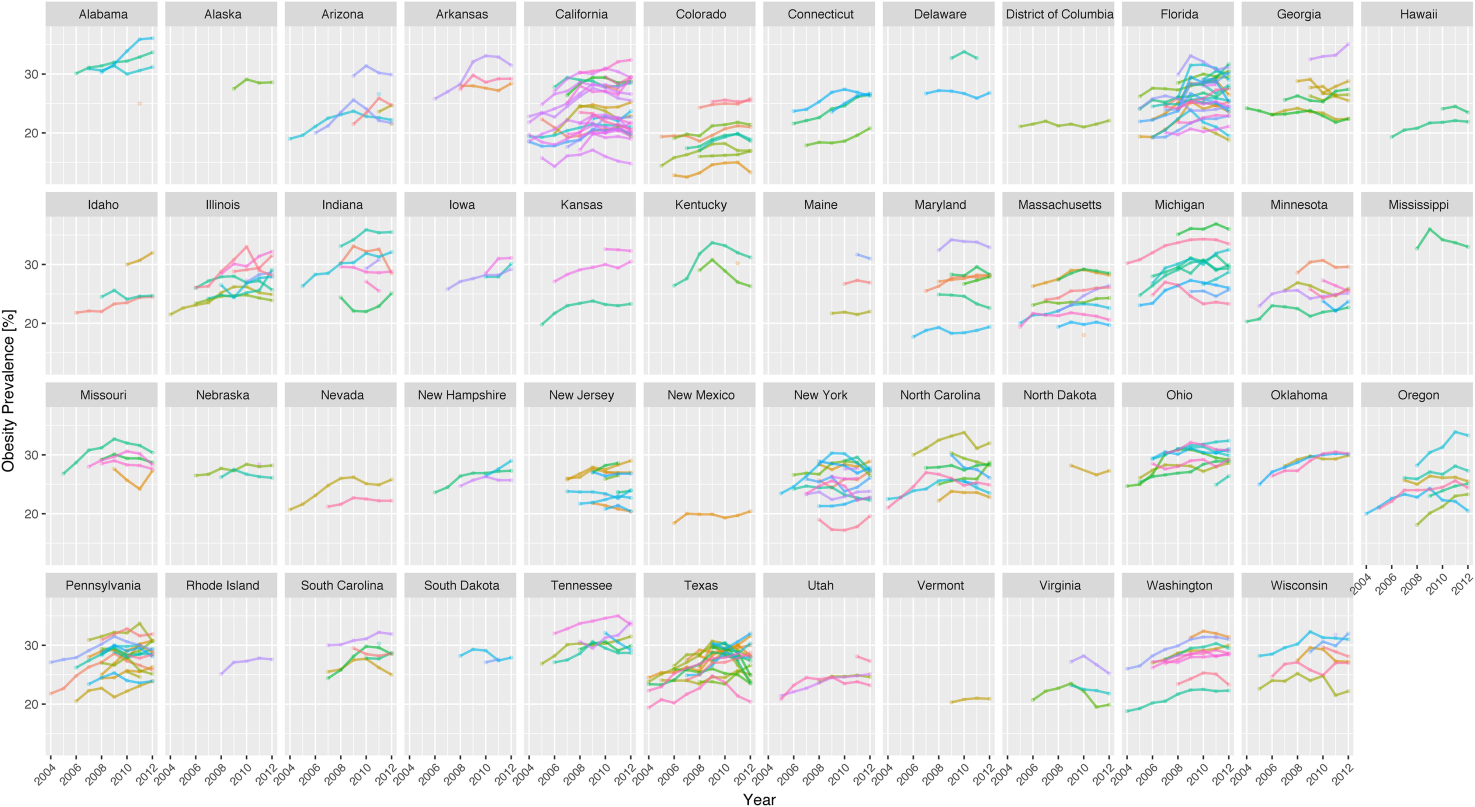
Trends of obesity prevalence levels as a function of time (2004–2012) for states and corresponding counties (presented as lines) in the US. We only report states and counties with at least 30 users bookmarking recipes in each of the counties for each year.

Figs 1 and 2 depict the linear trends in obesity prevalence over time at state and county level, respectively. The figures are derived from US Census data and to make the trends legible only counties where at least 30 users had bookmarked at least one online recipe were included. In Fig 1 each data point reflects the mean obesity prevalence within a county, while the linear line is an extrapolation of these data points to show the trends over time at state level.

**Fig 3:**
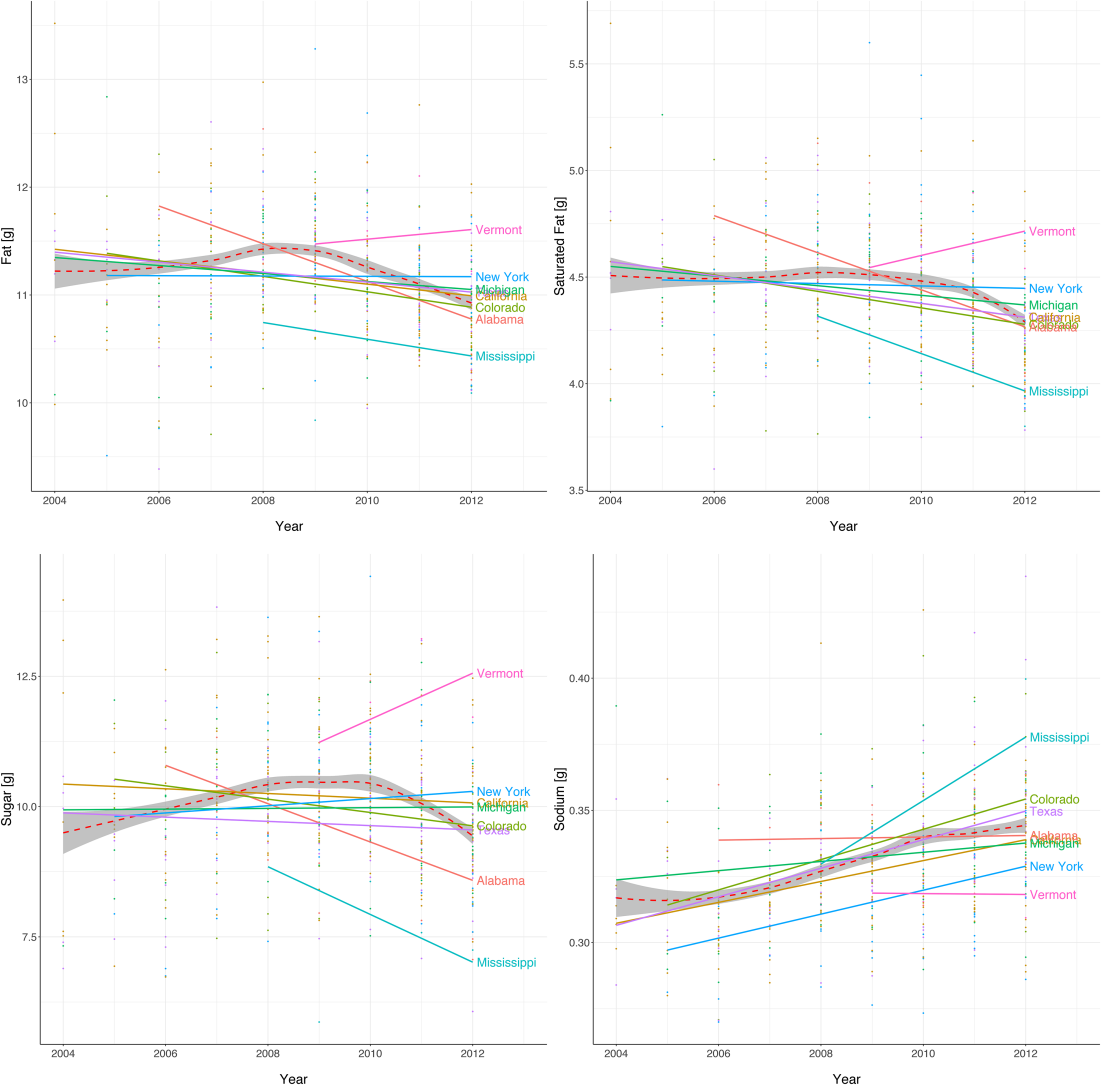
Trends for fat (top-left plot), saturated fat (top-right plot), sugar (bottom-left plot) and sodium (bottom-right plot) measured in 100g per recipe as a function of time (2004–2012) for the same selected US states as depicted in Fig 1. The general aggregated trend is shown with a dashed line.

**Fig 4:**
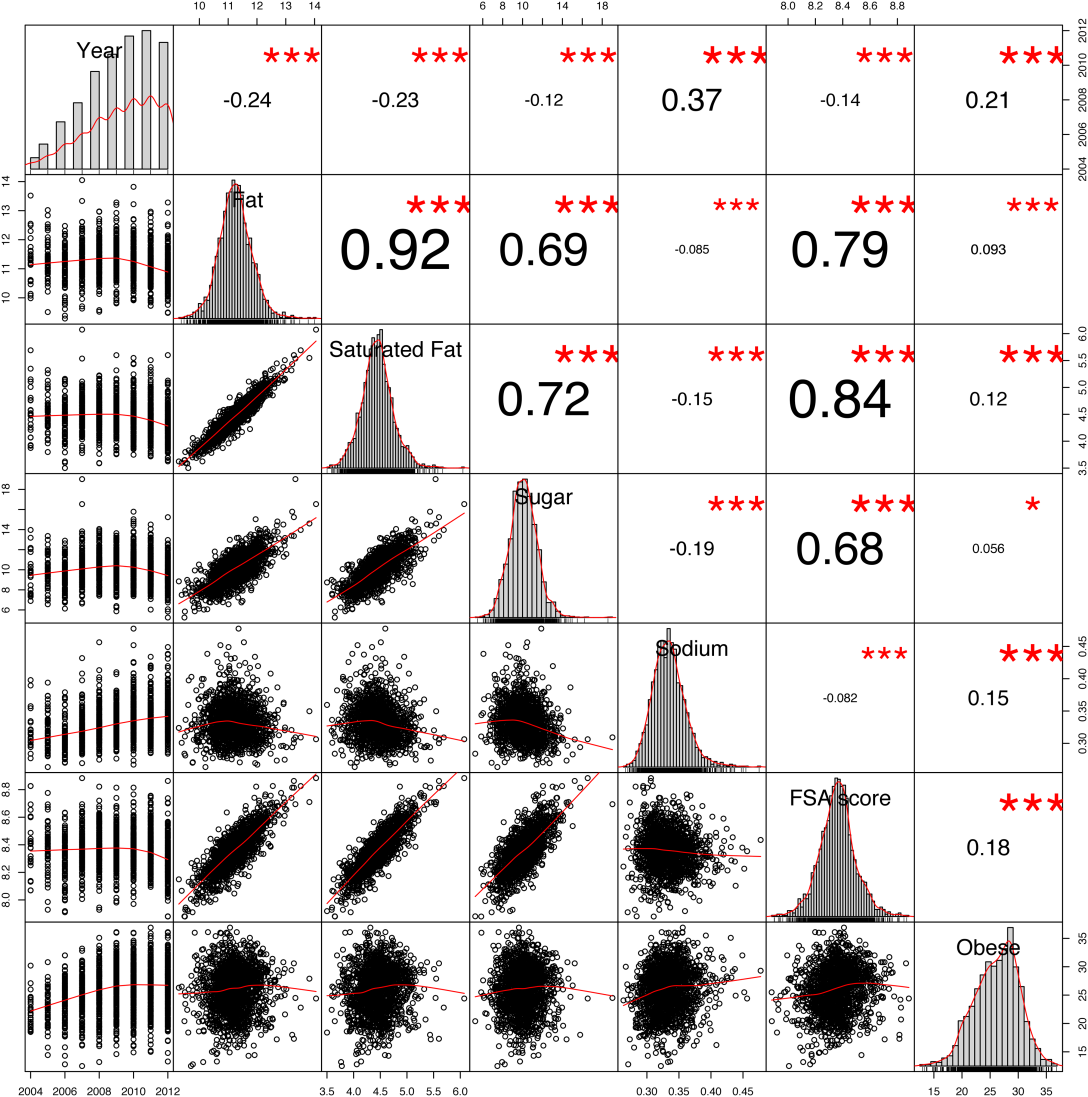
County-level correlations (spearman). Note: ***p < 0.001, *p < 0.05.

**Fig 5:**
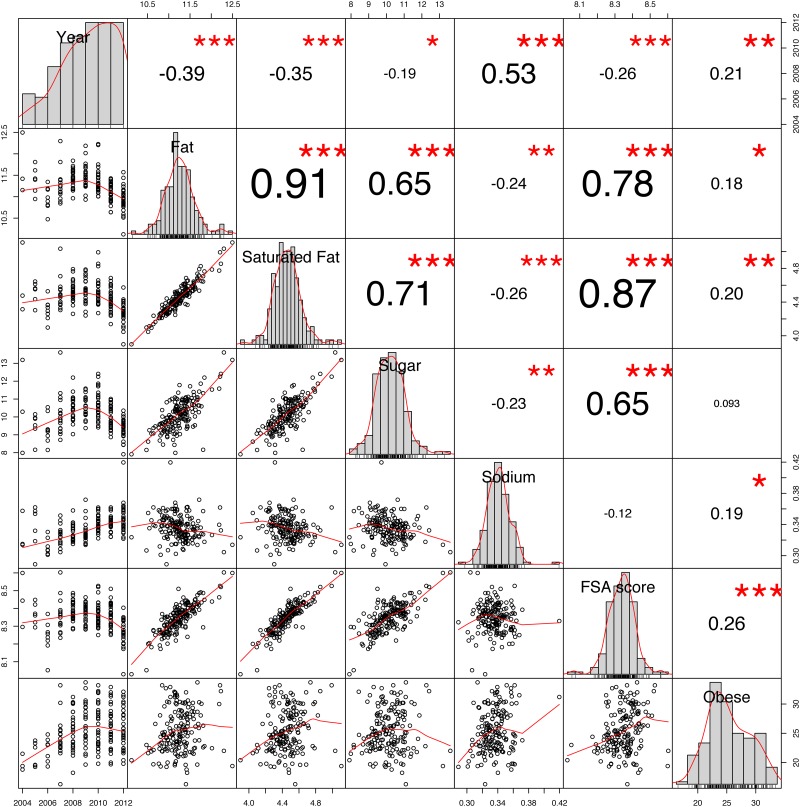
State-level correlations (spearman). Note: ***p < 0.001, **p < 0.01, *p < 0.05.

Additionally, Fig 3 shows the trends for fat (top-left plot), saturated fat (top-right plot), sugar (bottom-left plot) and sodium (bottom-right plot) as a function of time, for the same selected states as presented in Fig 1. What these plots show is that overall we see a gradual increase in fat and sugar content per 100g of food from 2004 up until some point between 2008 and 2010. After this, the aggregated quantity of these macro nutritional properties per 100g decreases. Similar trends are also observed in the correlations presented in the first columns of Figs 4 and 5. It is important to note that during this time period we also observe important differences in the trends for individual states, similar to what is shown in Fig 1 for obesity prevalence. The patterns for sodium and fat show different trends, with sodium showing a constant increase over time while saturated fat declines between the years 2008 and 2010 when the values are aggregated. Again, the trends are different for individual states, which is important for our modelling process.

Fig 1 provides evidence to justify our approach of fitting multilevel regression models with random intercept and random slope for each state if we observe their evolution over time. For instance, while the state of Michigan had an average obesity prevalence level of around 28% in 2004 and a steep growing trend until 2012, the state of Colorado had an initial average obesity level of only around 16% in 2004 and a less steep rate of growth. In addition, we have states without observations for every year, such as Mississippi and Vermont, which had enough data (at least 30 users per county each year in Allrecipes.com) only starting from 2008 and 2010, respectively.

These special characteristics of the dataset (differing temporal patterns at state and county levels, missing observations for counties for some and sometimes all years, and prevalence of datapoints for particular geographic regions (e.g. California and Texas)) are shown clearly in Fig 2. Multi-level regression models are robust and well suited to modeling datasets with these properties.

Following the methodology in [52, 53], we build models of increasing complexity: unconditional-means models, unconditional growth models and finally a model with random intercept and slope including nutritional properties as fixed factors.

In total we report four longitudinal multilevel regression models, which predict level of obesity. Each of these models can be expressed in a three-level equation such as:

$$\begin{array}{cc} Level1: & Obesity_{tij}=\pi_{0ij}+\pi_{1ij}\left(year\right)+\pi_{kij}\left(nutritionalVars_{k}\right)+\epsilon_{tij} \end{array}$$ (1a)

$$\begin{array}{cc} Level2: & \pi_{0ij}=\beta_{00j}+\beta_{01j}\left(county\right)+r_{0ij}\\ & \pi_{1ij}=\beta_{10j}+\beta_{11j}\left(county\right)+r_{1ij} \end{array}$$ (1b)

$$\begin{array}{cc} Level3: & \beta_{00j}=\gamma_{000}+\gamma_{001}\left(state\right)+u_{00j}\\ & \beta_{01j}=\gamma_{010}+\gamma_{011}\left(state\right)+u_{01j}\\ & \beta_{10j}=\gamma_{100}+\gamma_{101}\left(state\right)+u_{10j}\\ & \beta_{11j}=\gamma_{110}+\gamma_{111}\left(state\right)+u_{11j} \end{array}$$ (1c)

In the set of Eqs 1a–1c, the variable *Obesity*_*tij*_ identifies the obesity prevalence level—variable collected from census information—at county level and it is observed at time *t* for county *i* and state *j*. The model has random intercept *π*_0*ij*_ and also a random slope *π*_1*ij*_ for the coefficient *year*. Level 1 equation also contains the fixed effects of nutritional variables (*nutritionalVars*_*k*_) such as proteins and carbohydrates. We model them at level 1 since these measures vary every year. *ϵ*_*tij*_ corresponds to the error at level one, i.e., the variation in *Obesity*_*tij*_ data which is not explained by the model. Next, the *county* and *state* information is encoded in levels 2 and 3 within the random intercept *π*_0*ij*_ and the slope for *year*. More specifically, the intercept at level 1 is modeled at level 2 as a function of county *π*_0*ij*_, and similarly the coefficient *π*_1*ij*_ accompanying year. Finally, the intercepts and slopes of the aforementioned models are modeled as function of state (counties are located within states), where *β*_00*j*_ and *β*_01*j*_ are related to the random intercept at level 1, whereas *β*_10*j*_ and *β*_11*j*_ are related to the random slope of year at level 1. We use a diagonal variance structure, which is the default in the lmer package [54].

## Results

In this section we present the results of our analyses. First, the correlation analysis reveals not only temporal patterns, but also valid relationships between nutritional properties of bookmarked recipes and obesity. The results of the subsequent longitudinal multilevel regression analysis quantify more precisely the influence of each of the aforementioned dimensions (geographical, temporal), as well as nutritional factors (particularly “fat” and “sugar”, but also the combined FSA score) with respect to the levels of obesity.

### Correlation analysis

We performed the correlation analysis at two geographical levels, county-wise and state-wise, as shown in the correlation matrices in Figs 4 and 5.

The final row of each matrix depict how obesity incidence correlates with the other variables. Although there are significant correlations with different variables, we highlight three important observations. First, the two strongest correlations with obesity prevalence are time (i.e. year) and the FSA healthiness score of the recipes bookmarked by the users. At county level, the correlation between obesity prevalence and time (year) is *ρ* = 0.21, *p* < .001, whereas the FSA score shows a correlation of *ρ* = 0.18, *p* < .001 with obesity. At state level, a similar but stronger correlation between the FSA score and obesity is observed *ρ* = 0.26, *p* < .001. Second, we discover a correlation between the nutritional variables (Fat, Saturated Fat, Sugar and Sodium) of the recipes bookmarked with Obesity prevalence. These are nevertheless not as pronounced as the correlation between obesity and the FSA score, which combines all four variables in to a single health measure. The rho values range from *ρ* = 0.093 (Fat) to *ρ* = 0.15 (Salt) at county level and from *ρ* = 0.093 (Sugar) to *ρ* = 0.20 (Saturated Fat) at state level.

Finally, we observe a significant correlation between all variables studied, although on state level two variables show non-significant correlations. Multicollinearity can become a problem for the interpretation of regression models [55]. Correspondingly, in our analyses we employ a stepwise procedure to discard variables too correlated and keep only those with larger effect and without large correlation to other predictors.

### Multilevel regression analysis

We present the results of the multilevel regression analysis by presenting four of the models created. The modeling procedure followed the suggestion of several authors to build multilevel regression models by incrementally increasing the number of random and fixed effects and testing the model fitting using Akaike Information Criteria (AIC), Bayesian information Criteria (BIC), and likelihood-ratio test [52, 53]. We first checked the need for using multilevel models by comparing a null baseline model –fitted using a single intercept– against a multilevel model with random intercept which allowed the intercepts to vary across different counties/states.

A likelihood-ratio test between the baseline null model (*AIC* = 9363.07, *BIC* = 9373.92) and the multilevel model (*AIC* = 6796.61, *BIC* = 6818.30) shows that the second model fits the data significantly better (*χ*^2^(2) = 1285.25, *p* = 0), so we proceed with the multilevel regression analysis.

Table 2 summarizes four models for obesity which help to explain the effect of each factor. Model 1, an unconditional means model, accounts for the diversity of obesity only with the random effects based on geographical variables county and state. The random effect county:state has a *between-county/state* variance of 8.90, while the *between-state* variance is smaller, 4.87. Both variances are comparatively large with respect to a residual variance of only 1.79, what results in an intra-class correlation for county:state of *ICC*_*county*: *state*_ = 0.88 and for state of *ICC*_*state*_ = 0.31. These large intra-class correlations tell us that a multilevel model is necessary to explain the data (due to clustering effect within geographical regions) and also that a large amount of the variance of obesity is already explained by this simple model [56].

**Table 2:** Multilevel models for obesity. Models 3 and 4 incorporate a random intercept per county/state, a random slope for year, and fixed effects for the FSA score and Fat and Sugar. A likelihood ratio test shows significant differences between the models: Model 1 vs Model 2: *χ*^2^(5) = 585.64, *p* < 0.001; Model 2 vs Model 3: *χ*^2^(1) = 23.91, *p* < 0.001; Model 3 vs Model 4: *χ*^2^(1) = 14.67, *p* < 0.001. For the fixed effects, the number in parenthesis shows the standard error.

	Model 1	Model 2	Model 3	Model 4
*Variance Components*				
Var: county:State (Intercept)	8.90	9.01	8.84	9.02
Var: State (Intercept)	4.87	5.35	5.28	5.31
Var: Residual	1.79	0.97	0.96	0.94
Var: County:State Year		0.09	0.09	0.09
Cov: County:State (Intercept) Year		-0.28	-0.27	-0.28
Var: State Year		0.00	0.00	0.00
Cov: State (Intercept) Year		-0.04	-0.04	-0.04
*Fixed Effects*				
(Intercept)	26.56*** (0.39)	24.89*** (0.42)	14.27*** (2.20)	21.74*** (0.83)
Year		0.30*** (0.03)	0.31*** (0.03)	0.32*** (0.03)
FSA score			1.26*** (0.26)	
Fat/100g				0.19* (0.08)
Sugar/100g				0.08* (0.03)
AIC	6796.61	6226.47	6205.44	6200.72
BIC	6818.30	6275.28	6259.68	6260.38
Log Likelihood	-3394.31	-3104.23	-3092.72	-3089.36
Num. obs.	1675	1675	1675	1675
Num. groups: county:state	311	311	311	311
Num. groups: state	47	47	47	47

Note:

*** *p* < 0.001,

* *p* < 0.05

Model 2 adds a random intercept for the temporal variable *year* and no other fixed effects. This type of model is known as unconditional growth model [52]. Compared to Model 1, it has a smaller residual variance of 0.97. This drop in residual variance tells us that 45.7% of the within-county and within-state variation in obesity is systematically associated with linear time, considering $45.7\%=\frac{\left(1.79-0.97\right)}{1.79}$ [57]. Since the variable of time, *year*, was centered in 2004 = 0, this means that in 2004 the average obesity was 24.89% as observed in the significant intercept, *p* < 0.001. The coefficient associated to year is = 0.3, which means that every consecutive year the obesity level is increased by 0.3%. Model 2 has a significantly better fit than Model 1 tested with likelihood ratio test, Model 1 vs Model 2: *χ*^2^(5) = 585.64, *p* < 0.001.

Our third model, Model 3, incorporates fixed effects to Model 2. In particular it adds the FSA health score to the model. As shown in Table 2 the effect is significant, implying that for every additional unit of the FSA score, the the obesity level increases by 1.26. What is worth noting here is that this effect is stronger than the effect of time (Year) though lower than the impact of state/county. This model has a significantly better fit than Model 2, assessed by the decreased in *AIC*, *BIC* and by the likelihood ratio test, Model 2 vs Model 3: *χ*^2^(1) = 23.91, *p* < 0.001.

Our fourth and final model, Model 4, includes nutritional variables. We tested all the nutritional variables, but we found that adding “Fat” and “Sugar” produced the best fit (again employing a stepwise procedure). The effect is rather small compared to the variance already explained by the geographical and temporal dimensions, but it is still significant and positive, implying that for every additional unit of fat/100g and sugar/100g of the recipe, the obesity level increases by 0.19% (for fat) and 0.08% (for sugar). This last model has a significantly better fit than Model 3, assessed by the decreased *AIC* and by the likelihood ratio test, Model 3 vs Model 4: *χ*^2^(1) = 14.67, *p* < 0.001.

## Discussion

The models presented in the previous section confirm temporal, geographical and nutritional relationships, which help to explain the variance in obesity incidence as recorded via US census data.

Previous work in our field has revealed patterns in interactions with information systems, such as submitted search queries and the content social-media posts, which correlate with health-related problems. Our findings confirm that interactions with the online food portal Allrecipes.com can be similarly insightful, in this case by showing how the nutritional properties of bookmarked recipes relate to incidence of obesity in the US. Nevertheless, the modeling process highlights that other factors—temporal and geographical—are also very important and explain large portions of the variance in obesity rate. Our analyses show that when using these kinds of data for epidemiological purposes, it is vital to consider these additional factors in the process.

There has been great debate and no little controversy in the nutritional science community, as well as the media coverage of published research [58–60] regarding the role of the metabolic effects of dietary components. Much of the discussion revolves around whether sugars or fats play the greatest role in explaining health problems [38, 61–63]. The blame for rising obesity rates seems to shift regularly from fats to sugars and then back again [64]. The guidelines from the WHO recommend that intake of sugar- and energy-dense, fatty foods should be limited and intake of foods with high fiber content increased to reduce the likelihood of suffering from obesity [37]. We found that both the average fat and sugar content of bookmarked recipes were useful predictive features for obesity rates with fat content explaining slightly more of the variance than sugar (model 4). Disappointingly, including fiber as a predictive feature in our models did not improve the fit to the data.

When combining geographical, temporal and nutritional features from interaction patterns the multi-level regression models offer a good fit to the data. This indicates that such information could be a valuable asset in monitoring health trends between census publication dates.

At the same time, the temporal trends revealed in the modeling process are concerning because they suggest that obesity rates, which are already very bad and costly for our society, are only going to get worse over time. It is therefore important for researchers and practitioners to investigate ways in which this kind of technology cannot only monitor user health, but actively change it for the better. That is by including algorithmic [65, 66] and interface features [67, 68] in food portals, such as Allrecipe.com which lead users to consume healthier recipes, technology may be able to positively impact obesity rates. This may or may not influence the utility of such services as a epidemiological tool.

## Conclusions

This article has presented the results of an investigation to determine the extent to which interaction data from the online food portal Allrecipes.com can explain the variance in obesity rates across the US. Given that the site has a large user population spread across the country and there are known links between food consumption and obesity incidence, the interaction data might be useful in providing readily accessible and up-to-date information on obesity incidence.

### Findings

Our findings can be summarised as follows:

- We demonstrate significant and meaningful (i.e. sensibly interpretable) relationships between the nutritional properties of bookmarked recipes (sugar content, fat content and a combined FSA-score for recipes) and obesity incidence.
- As our dataset spans a 9 year time period we show the stability of such data as predictors.
- We show that temporal patterns are also important with obesity rate increasing overall over time. We do acknowledge, however, that there are counties and states for which this is not the case.
- The geographical differences in obesity patterns mean that it is also important to account for these in the modeling process.
- The good fit achieved by our models suggests that combining interaction data, geographical data and temporal data can be a useful in monitoring obesity incidence.

### Limitations

There are several limitations to the analyses we have presented and we wish to acknowledge these directly.

- The analyses relate only to the United States and in particular to one food portal—Allrecipes.com. There is no evidence that the findings would be repeated with other websites or in other countries.
- The user demographics of Allrecipes.com are skewed in general towards wealthy and densely populated areas. The socio-economic factors relating to obesity are well-documented—higher rates are found among groups with lower educational and income levels, among racial and ethnic minorities, and in high-poverty areas [64]. Although the modeling approaches applied are typically robust to these kinds of issues, the data available may have restricted the quality of the fit achieved.
- We restricted our focus to four micro/macro-nutritional components (fat, saturated-fat, sugar and sodium) as well as the FSA metric combining these. Other interesting variables we may have investigated include the WHO metric [48], which accounts for additional properties such as Fiber and Carbohydrates. The USDA Healthy Eating Index (HEI) [69] also encompasses different aspects in the model such as vegetables, fruits, etc.

### Future work

We are currently extending and planning to extend our work in numerous ways:

- We are extending our models to include other interactive features (ratings, comment sentiment etc.) to establish if these offer better or additional insight into obesity incidence. In addition, we will investigate the use of nonlinear hierarchical models [70] to better account for the not strictly linear nature of the relations between nutritional elements and outcome variables such as obesity or diabetes.
- We are also examining the WHO [66] and HEI [69] metrics to see if these offer additional utility.
- We are investigating the possibility of repeating our analyses with other data sources. In particular we hope to repeat the analyses for data in a different country.
- We hope to investigate whether interaction data from Allrecipes.com can be combined with other interaction sources, such as longitudinal social media content. The intuition here is that these sources may offer different and complementary insight.
- We plan to develop more complex models to investigate the relation of climate. The intuition being that people living in locations with colder climates may be prone to eating more caloric food than people in warmer regions, without this implying larger obesity or diabetes levels. Analyzing this relation more closely might help understanding the effect of geographical factors.

## Supporting information

S1 File Allrecipes.com dataset. Averaged Allrecipes.com nutritional data for bookmarked recipes and corresponding health statistics for US counties and states for the time period 2004 to 2012.
(CSV)
